# Selection on the wing in *Heliconius *butterflies

**DOI:** 10.1186/1471-2156-12-31

**Published:** 2011-03-28

**Authors:** Delphine Legrand, Virginie M Stevens, Michel Baguette

**Affiliations:** 1 UMR 7204, MNHN-CNRS-UPMC, Laboratoire CERSP, 75005 Paris, France; 2 CNRS USR 2936 Station d'Ecologie Expérimentale du CNRS à Moulis, 09200 Moulis, France; 3 FNRS-F.S.R. & Université de Liège, Unité de Biologie du Comportement, Liège, Belgium

## Abstract

**Asbtract:**

To what extent population structure favours the establishment of new phenotypes within a species remains a fundamental question in evolutionary studies. By reducing gene flow, habitat fragmentation is a major factor shaping the genetic structuring of populations, favouring isolation of small populations in which drift may rapidly change frequencies of new variants. When these variants provide advantages to individuals, the combined effect of selection and drift can lead to rapid shifts in phenotypes. In a study published in *BMC Genetics*, Albuquerque de Moura *et al. *asked whether such a general pattern of population structure can be observed in *Heliconius *species, which could have strong implication in the evolution of colour pattern diversification in these butterflies. In this commentary we discuss the potential roles of these three processes (drift, selection and dispersal) on the evolution of *Heliconius *wing patterns in regard to the findings of a common fine-scale population structure within the co-mimetic species *H. melpomene *and *H. erato*. Indeed, a general pattern of population subdivision in the history of these two species may have provoked the major phenotypical shifts observed in their wing colour patterns. The suggestion that coupled environmental pressures (counter-selection of dispersal and selection on co-evolved traits) could be responsible for identical genetic differentiation profiles in *H. erato *and *H. melpomene *clearly merits further investigations using both detailed population genetic (including landscape genetic) and ecological studies.

## 

Rapid changes of phenotypes can be observed in small genetically isolated population because drift may enhance the rate of fixation of new variants. As a result, the long-term evolution of traits with ecological importance is thought to be tightly linked to the historical population structure of species for which strong genetic isolation is a common feature. Of course, species living in highly fragmented landscapes are more prone to exhibit strong genetic subdivision because gene flow will tend to be reduced in patchily distributed habitats.

The study of Albuquerque de Moura *et al. *[1] published in *BMC Genetics *aimed at determining if such a general pattern of population structure can be observed in *Heliconius *species, which could have strong implications in the evolution of colour pattern diversification in these butterflies. Using a panel of genetic markers, they effectively found a widespread genetic differentiation of populations on unusually small geographic distances in the Mullerian co-mimetic butterflies *Heliconius erato *and *H. melpomene*, but no isolation by distance [1]. Populations were sampled in Brazil's Atlantic Forest, a biodiversity hotspot that is now highly fragmented after 500 years of human disturbance. The low connectivity of this fragmented landscape for butterflies, resulting in a steep decrease in dispersal, and hence gene flow among populations, is proposed as the main driver of the observed genetic differentiation. Dispersal depression along fragmentation gradients and subsequent population differentiations has indeed been recorded in butterflies [2,3]. However, given the huge dispersal abilities of butterflies [4], such a high genetic differentiation of populations distant from 5 km and less is intriguing. It has particularly been established that average dispersal distance in *Heliconius *butterflies ranges from 3 to 10 km [5,6]. Thus, the genetic differentiation as described between the neighbouring populations of Albuquerque de Moura *et al. *study [1] could not be due to a simple dispersal-limited explanation. We thus suggest that other factors than dispersal counter-selection due to prohibitive costs could be at work in this study system.

The use of different marker types to infer genetic population structure has now become a widespread and powerful strategy. Although dominance effects, as well as mutational processes, involve fundamental differences in marker evolution, they should all reflect the same population history [7], provided a sufficient number of loci are used. When effective population sizes are small and contrasted in the study system, genetic drift enhances the accumulation of heterogeneity within the genome, highlighting the necessity to adopt a wide locus-sampling strategy [8], more efficiently obtained with dominant markers [9]. In such cases, it is recommended to use 10 times as many loci for dominant compared to codominant markers [8]. It is noteworthy that Albuquerque de Moura *et al. *[1] have genotyped individuals with three kinds of marker. Indeed, they used 5 and 7 microsatellites, 81 and 15 mitochondrial SNPs respectively for *H. erato *and *H. melpomene *and 1144 AFLP for both species to properly infer the genetic structuring of each species. If all marker types revealed the presence of a fine-scale population structure within both species, inter-marker patterns within species were not correlated (Mantel tests non significant at the 5% level). This discrepancy between mitochondrial and nuclear markers may reflect different dispersal behaviours between males and females as proposed by the authors. However, the discrepancy between genetic structure inferred from microsatellites and AFLP markers is probably the result of an insufficient number of microsatellite loci preventing high enough statistical power, which is especially true when populations have not reached genetic equilibrium [8]. In contrast, the huge number of AFLP used in this study should be sufficient to properly sample genome diversity.

Thus, we think that the more reliable results concerning the nuclear genome are those obtained with AFLP. Comparing patterns of genetic differentiation obtained with AFLP, we found a strong correlation between *H. erato *and *H. melpomene *(Mantel test: R = 0.55, p = 0.012). In other words, pairs of populations harbour similar genetic divergence levels in the two species. We suggest that such strong parallel patterns of genetic isolation can be observed when species face the same environmental pressures.

An interesting point is the possibility of a convergent pattern of genetic isolation between co-distributed *Heliconius *species due to selection pressures acting on similar genomic regions. Among potential candidate characters on which selection could generate such genomic patterns, the coloration of wing patterns, which serves as a warning signal for predators, is noteworthy. It has been shown that wing patterns in co-mimetic *Heliconius *species are driven by several homologous genomic regions functioning as "supergenes" [10-12]. Those regions under long-term selection have evolved independently in *Heliconius *lineages (among which *H. melpomene *and *H. erato*) with parallel changes in gene regulation across co-mimetic species [13,14]. They provide a remarkable example of convergent evolution. Such genetic determinism with convergent selection at several distant loci could account for the similar genetic differentiation obtained with AFLP in the two species, as AFLP are presumably scattered across the genome. However, all populations of *H. melpomene *and *H. erato *share the same wing pattern phenotype in the studied region (Albuquerque de Moura *et al. *pers. com.). Nonetheless, we encourage a detailed morphological analysis that could reveal subtle co-evolved changes in the wing colour patterning of populations in both species. Besides, we propose the careful investigation of ecological mechanisms that would allow the maintenance of the observed genetic differentiation, like assortative mating between individuals of the same populations or local specialization on particular host plants. Indeed, the genetic determinism of such ecological mechanisms is probably complex.

Overall, it seems that adaptive responses under particular environmental constraints may involve the same genomic regions in populations of co-distributed *Heliconius *species. This finding could be of great importance when trying to retrace the evolutionary history of *Heliconius *species. As mentioned by the author, the additional effect of drift in these small populations of *Heliconius *can rapidly increase the frequency of new benefit alleles, enhancing the rate of change in the genetic structuring and phenotypes in populations. In what measure drift and selection have influenced shifts in *Heliconius *wing patterns remains an exciting question that needs to be raised in the context of habitat fragmentation, taking advantage of the new advances in landscape genetics.

To conclude, we think that an additive or an interaction effect of (1) dispersal counter-selection in response to habitat fragmentation and (2) selection on important ecological traits (including wing traits and ecological mechanisms maintaining population differentiation) might be responsible for the genetic structuring we observe here. In this sense, the study of Albuquerque de Moura *et al. *[1] is the first step to our understanding of the interplay between landscape and adaptive traits shaping the genetic structure of *H. melpomene *and *H. erato *populations at a local scale. Finally, extending this population genetic study using selected loci responsible for wing pattern expression needs to be considered to determine the putative role of 'selection on the wing' in this system, and to go further in our comprehension of the long-term processes underlying co-mimicry in *Heliconius*.

## Authors' contributions

DL, VMS and MB conceived of the manuscript, DL wrote the manuscript, all authors read and approved the final manuscript.
